# Beyond the apnea–hypopnea index: alternative diagnostic parameters and machine learning solutions for estimation of sleep apnea severity

**DOI:** 10.1093/sleep/zsab134

**Published:** 2021-09-13

**Authors:** Timo Leppänen, Sami Myllymaa, Antti Kulkas, Juha Töyräs

**Affiliations:** 1 Department of Applied Physics, University of Eastern Finland, Kuopio, Finland; 2 Diagnostic Imaging Center, Kuopio University Hospital, Kuopio, Finland; 3 School of Information Technology and Electrical Engineering, The University of Queensland, Brisbane, Queensland, Australia; 4 Department of Clinical Neurophysiology, Seinäjoki Central Hospital, Seinäjoki, Finland; 5 Science Service Center, Kuopio University Hospital, Kuopio, Finland

We have read with great interest the paper “Metrics of Sleep Apnea Severity: Beyond the AHI” written by Malhotra et al. [1], which was published in the *Sleep* on March 9, 2021. The authors presented research-based recommendations to enhance and optimize the diagnosis and severity estimation of obstructive sleep apnea (OSA). The authors also presented alternative metrics, such as the Hypoxic Burden and the apnea–hypopnea event duration, for estimation of OSA severity. They discussed the potential future directions of OSA diagnostics such as machine learning and wearable technologies. While we certainly agree that this is an important topic and the apnea–hypopnea index (AHI) is not the best metric to describe the severity of OSA, at the same time, we wonder how the authors have completely missed a vast amount of relevant research papers that have been published during the past decade. Albeit the authors state that the paper is not intended to be “an exhaustive review of the available literature,” the paper lacks numerous highly relevant references, which are very strongly related to the topic. For example, our research group has published over 50 scientific papers on this topic (20 most relevant ones listed in Table 1). However, none of these scientific papers were cited in the Research Statement.

**Table 1.** Twenty selected scientific articles related to alternative metrics for obstructive sleep apnea diagnosis, wearable technologies, and machine learning solutions published by our research group

Alternative diagnostic metrics	
Kainulainen S, Duce B, Korkalainen H, et al. Severe desaturations increase psychomotor vigilance task-based median reaction time and number of lapses in obstructive sleep apnoea patients.	*Eur Respir J*. 2020; 55 (4): 1901849
Kainulainen S, Töyräs J, Oksenberg A, et al. Severity of desaturations reflects OSA-related daytime sleepiness better than AHI.	*J Clin Sleep Med*. 2019; 15 (8): 1135–1142
Leppänen T, Kulkas A, Oksenberg A, et al. Differences in arousal probability and duration after apnea and hypopnea events in adult obstructive sleep apnea patients.	*Physiol Meas*. 2018; 39 (11): 114004
Kulkas A, Duce B, Leppänen T, et al. Gender differences in severity of desaturation events following hypopnea and obstructive apnea events in adults during sleep.	*Physiol Meas*. 2017; 38 (8): 1490–1502
Leppänen T, Kulkas A, Duce B, et al. Severity of individual obstruction events is gender dependent in sleep apnea.	*Sleep Breath*. 2017; 21 (2): 397–404
Leppänen T, Särkkä M, Kulkas A, et al. RemLogic plug-in enables clinical application of apnea–hypopnea index adjusted for severity of individual obstruction events.	*J Med Eng Technol*. 2016; 40 (3): 119–126
Myllymaa S, Myllymaa K, Kupari S, et al. Effect of different oxygen desaturation threshold levels on hypopnea scoring and classification of severity of sleep apnea.	*Sleep Breath*. 2015; 19(3): 947–954
Muraja-Murro A, Kulkas A, Hiltunen M, et al. Adjustment of apnea–hypopnea index with severity of obstruction events enhances detection of sleep apnea patients with the highest risk of severe health consequences.	*Sleep Breath*. 2014; 18 (3): 641–647
Kulkas A, Tiihonen P, Julkunen P, et al. Novel parameters indicate significant differences in severity of obstructive sleep apnea with patients having similar apnea–hypopnea index.	*Med Biol Eng Comput*. 2013; 51 (6): 697–708
Kulkas A, Tiihonen P, Eskola K, et al. Novel parameters for evaluating severity of sleep disordered breathing and for supporting diagnosis of sleep apnea–hypopnea syndrome.	*J Med Eng Technol*. 2013; 37 (2): 135–143
Wearable sleep technologies	
Kalevo L, Miettinen T, Leino A, et al. Effect of sweating on electrode-skin contact impedances and artifacts in EEG recordings with various screen-printed Ag/AgCl electrodes.	*IEEE Access*. 2020; 8: 50934–50943
Miettinen T, Myllymaa K, Hukkanen T, et al. Home polysomnography reveals a first-night effect in patients with low sleep bruxism activity.	*J Clin Sleep Med*. 2018; 14 (8): 1377–1386
Miettinen T, Myllymaa K, Westeren-Punnonen S, et al. Success rate and technical quality of home polysomnography with self-applicable electrode set in subjects with possible sleep bruxism.	*IEEE J Biomed Health Inform*. 2018; 22 (4): 1124–1132
Miettinen T, Myllymaa K, Muraja-Murro A, et al. Screen-printed ambulatory electrode set enables accurate diagnostics of sleep bruxism.	*J Sleep Res*. 2018; 27 (1): 103–112
Myllymaa S, Muraja-Murro A, Westeren-Punnonen S, et al. Assessment of the suitability of using a forehead EEG electrode set and chin EMG electrodes for sleep staging in polysomnography.	*J Sleep Res*. 2016; 25 (6): 636–645
Machine learning	
Korkalainen H, Aakko J, Duce B, et al. Deep learning enables sleep staging from photoplethysmogram for patients with suspected sleep apnea.	*Sleep*. 2020; 43(11): zsaa098
Nikkonen S, Korkalainen H, Kainulainen S, et al. Estimating daytime sleepiness with previous night electroencephalography, electrooculography, and electromyography spectrograms in patients with suspected sleep apnea using a convolutional neural network.	*Sleep*. 2020; 43 (12): zsaa106
Kainulainen S, Töyräs J, Oksenberg A, et al. Power spectral densities of nocturnal pulse oximetry signals differ in OSA patients with and without daytime sleepiness.	*Sleep Med*. 2020; 73: 231–237
Korkalainen H, Aakko J, Nikkonen S, et al. Accurate deep learning-based sleep staging in a clinical population with suspected obstructive sleep apnea.	*IEEE J Biomed Health Inform*. 2020; 24 (7): 2073–2081
Nikkonen S, Afara IO, Leppänen T, et al. Artificial neural network analysis of the oxygen saturation signal enables accurate diagnostics of sleep apnea.	*Sci Rep*. 2019; 9 (1): 13200

First, the authors did not mention the Desaturation Severity parameter [2] when talking about the Hypoxic Burden. Instead, the authors claimed that “now investigators have captured the area under the oxyhemoglobin saturation curve as a metric of hypoxic burden” referring to the paper by Azarbarzin et al. [3] published in 2019. However, the idea of calculating the area of blood oxygen desaturation events has been introduced many years before that paper [2]. In addition, a letter to editor [4] of the *European Hearth Journal* was published related to the paper by Azarbarzin et al. [3], in which an incomplete literature review was criticized; they also missed the previously published papers related to the calculation of area under the desaturation events. Furthermore, when Malhotra et al. discussed the apnea–hypopnea event duration they stated that “Event duration has been quantified by Butler et al.” [5]. Albeit being true, the paper by Butler et al. [5] is most certainly not the first one presenting the idea of taking apnea and hypopnea event duration into account in the severity estimation of OSA. At least Kulkas et al. introduced this idea already in 2013 [2].

Second, we agree with the authors that novel wearable technologies have a great potential to enhance the diagnosis of sleep disorders and evaluation of sleep quality in the future. Albeit there is lots of ongoing research related to, for example, simplification of devices intended for diagnosis of OSA, many of these novel approaches are not yet accurate and reliable enough compared to conventional polysomnography or home sleep apnea test devices. Furthermore, certain devices used in sleep research lack clinical validation according to medical device regulations and are considered consumer-grade devices. However, the authors discussed wearable technologies on a very general level providing an example on only one device while citing the paper written by de Zambotti et al. [6] presenting a much broader discussion on the available wearable sleep technologies. The authors could have even mentioned devices like Fitbit, Jawbone, Misfit, and ŌURA ring discussed by de Zambotti et al. [6]. Leaving the most promising technologies out of the discussion in the review paper does not provide the readers the correct overall picture of what is happening in the field of wearable sleep technology.

Third, when the future directions and machine learning were discussed, it was again very surprising to see how many relevant research papers were not cited. Just to pinpoint a couple, Phan et al. [7] and Gutiérrez-Tobal et al. [8] have done lots of research involving machine learning. They have, for example, introduced deep learning solutions for the classification of sleep stages from polysomnographic signals [7] and estimated OSA severity class from a blood oxygen saturation signal [8]. In addition, we have also recently published several research papers on how to utilize machine learning to analyze polysomnography data. In these publications, deep learning methods were developed to estimate the value of AHI from blood oxygen saturation signal [9] and automatically classified sleep stages from a single electroencephalography signal [10]. Mirroring the results and topics of the papers by us (Table 1) and others [7, 8] to the topic of the Research Statement, the authors’ selection of the references is not appropriate.

To conclude, the topic of the Research Statement is highly relevant and timely tackling the very important issue. However, as the aim of the Research Statement was to discuss novel polysomnography metrics and future directions, and provide “recommendations for research needed to optimize OSA diagnostics,” we feel that the literature review included in the Research Statement is very incomplete. This is worrying especially considering the influential positions of the authors in the field of sleep medicine. Furthermore, we feel that the pioneering work should always be respected in science. We believe that in the spirit of fair play and collaboration, researchers worldwide have great opportunities to make major technological advances in the field of sleep medicine.
